# When emotions hurt: negative interpretations of bodily signals and interoceptive difficulties in fibromyalgia

**DOI:** 10.1007/s00426-026-02341-2

**Published:** 2026-07-02

**Authors:** Aleksandra M. Herman, Julia Szczawińska, Carolyn Berryman, Tasha R. Stanton

**Affiliations:** 1 Laboratory of Brain Imaging, Nencki Institute of Experimental Biology of the Polish Academy of Sciences, Pasteur 3 St, Warsaw, Poland; 2 IIMPACT in Health, Adelaide University, Adelaide, South Australia Australia; 3 Persistent Pain Research Group, Hopwood Centre for Neurobiology, Lifelong Health Theme, South Australian Health and Medical Research Institute (SAHMRI), Adelaide, South Australia Australia; 4 Brain Stimulation, Imaging and Cognition Research Group, College of Health, Adelaide University, Adelaide, SA Australia; 5 Hopwood Centre for Neurobiology, South Australian Health and Medical Research Institute, Adelaide, SA Australia; 6 Women’s and Children’s Hospital, Adelaide, SA Australia

## Abstract

People with fibromyalgia often demonstrate hypersensitivity to benign bodily signals and misattribute non-noxious physical signals as pain, suggesting alterations in body-brain communication and interoception. However, little is known about whether altered body-brain communication may extend to misattribution of emotion as pain. Here, using a cross-sectional design, we aimed to investigate how individuals with fibromyalgia perceive, identify, and interpret emotions and other bodily sensations, including pain, compared to healthy controls. Across two independent studies, individuals with fibromyalgia and age- and gender-matched pain-free controls completed the emBODY task, which assesses the topography of bodily sensations experienced for different states (emotional states, neutral state, pain- and physiology-related states, fatigue). Additionally, participants completed assessments of alexithymia, bodily sensation interpretation, and interoception. In both studies, linear discriminant analysis, performed to test whether different emotions and states are associated with statistically distinct bodily patterns, indicated comparable classification accuracy of body sensation maps in the fibromyalgia group and controls. However, compared with controls, the fibromyalgia group showed higher levels of alexithymia, higher awareness of bodily signals, and more negative interpretation of ambiguous bodily sensations in daily life, together with greater self-reported interoceptive difficulties. Our findings support an amplified perception of bodily signals coupled with interoceptive difficulties in people with fibromyalgia. This raises the possibility that interventions that target perceptual retraining, and re-interpretation of bodily sensations might decrease the impact of pain and promote engagement in everyday activities for people with fibromyalgia.

**Supplementary Information:**

The online version contains supplementary material available at https://doi.org/10.1007/s00426-026-02341-2.

## Public significance statement

People with fibromyalgia often experience pain even when there is no clear physical cause. This research shows that they may have increased perception of certain bodily sensations, and are more likely to interpret unclear body signals as negative or painful. Understanding these differences in how the body and brain communicate may help develop new treatments that improve daily functioning and quality of life by retraining how bodily sensations are perceived and interpreted.

**Supplementary Information:**

The online version contains supplementary material available at https://doi.org/10.1007/s00426-026-02341-2.

## Highlights

Fibromyalgia shows amplified bodily awareness and negative signal interpretation.

Interoceptive difficulties occur despite no differences in performance in the Heartbeat Tracking Task.

Perceptual retraining may reduce pain-related distress and improve functioning.

**Supplementary Information:**

The online version contains supplementary material available at https://doi.org/10.1007/s00426-026-02341-2.

## Introduction

Fibromyalgia (FM) is characterised by widespread pain, fatigue, and cognitive symptoms (Wolfe et al., 2016) and affects around 2%–8% of the global population, predominantly women (Soroosh, 2024). The pain and other symptoms of fibromyalgia are considered to arise from nociplastic mechanisms; that is, from altered central processing of nociception in the absence of clear evidence of tissue or nervous system damage (Trouvin and Perrot 2019; Kosek et al., 2016). In nociplastic pain, disruption in body-brain communication is thought to occur, whereby information coming from the body is “misread” or “misattributed”, resulting in experiences that do not match the initiating stimulus, such as allodynia (pain in response to light touch) and/or hypersensitivity (heightened pain to a mild stimulus). In this way, the adaptive features inherent to acute pain (alerting individuals to potential harm, promoting protective behaviours) (Craig, 2003) become maladaptive, with the potential for over-protection, hypervigilance, and avoidance behaviour (Moseley and Vlaeyen, 2015), all of which relate to suboptimal clinical outcomes (Vlaeyen & Linton, 2000).

Differentiating between various physiological states and emotions is critical for adaptive behaviour and decision-making. However, individuals with chronic pain, such as fibromyalgia, frequently exhibit altered perception and interpretation of bodily signals (i.e. interoception) (Khalsa et al., 2018; Duschek et al., 2017; Schmitz et al., 2021), coupled with generalised hypersensitivity (Ceko et al. 2012; Berryman et al., 2021), difficulty identifying and describing emotions (Sayar et al., 2004) and changes in affective processing (Boone, 2007). These distortions can result in hypersensitivity to benign bodily cues, misattribution of physical sensations as pain, and maladaptive behaviours prioritising immediate relief over long-term well-being. Moreover, pain-related fear, negative expectations and attributions, and catastrophising can increase the intensity of subjective pain, and increase activation of pain-related circuits in the brain (Gracely et al., 2004). These processes may also be central to pain chronification by fuelling interoceptive hypervigilance and avoidance behaviour (Pinto et al., 2023). In this context, we use the term “bodily confusion” as a descriptive, integrative construct referring to reduced differentiation between bodily and emotional states at the level of subjective experience and behavioural reporting, rather than as a distinct theoretical mechanism (Herman et al., 2024).

Recent methodologies, such as the emBODY tool (Nummenmaa et al., 2014, 2018), have enabled evaluation of one’s ability to differentiate between physiological states and emotions. Participants mark areas on a human figure where they notice changes in bodily sensations during certain physiological states or emotions, thus creating a bodily sensation map (BSM). BSMs are consistent across cultures and languages (Volynets et al., 2020; Herman et al., 2022), and may be diagnostically informative as they differ between healthy controls and people with schizophrenia (Torregrossa et al., 2019) and depression (Lyons et al., 2021), for example. Previous work using the emBODY tool also revealed significant differences between healthy individuals and clinical pain populations, with the latter exhibiting altered bodily representations of emotion (Ojala et al., 2023). These findings highlight the interplay between pain, emotional processing, and somatic awareness, underscoring the need to investigate these aspects, given their potential contribution to behaviour and potentially quality of life. Yet to date, there has been little consideration of the differentiation of bodily sensations and emotions (or lack thereof) in people with chronic pain, specifically fibromyalgia, and whether this is altered relative to people without pain.

Across two studies with independent samples, we used the emBODY tool to explore the differentiation of emotions and physiological states in individuals with fibromyalgia, relative to pain-free controls. Additionally, we investigated how individuals with fibromyalgia sense and interpret various bodily sensations in everyday life (interoception), as well as their perceived ability to differentiate and name distinct emotions (alexithymia). We hypothesised that individuals with fibromyalgia would exhibit heightened perception of daily bodily sensations, accompanied by a diminished ability to differentiate between them and a tendency to interpret them more negatively. By examining these aspects of bodily sensations perception and interpretation, we aimed to deepen the understanding of the interplay between emotional and somatic awareness in fibromyalgia, offering a basis for targeted novel therapeutic interventions for this challenging condition.

## Study 1

### Methods

We conducted a cross-sectional study involving one in-person testing session at the University of South Australia (UniSA) that was undertaken between September 2023 and July 2024. All procedures were approved by UniSA’s Human Research Ethics Committee (Ethics Protocol 204754).

#### Participants

This study was a part of a larger project on decision-making in fibromyalgia described elsewhere (Herman et al., 2025). Based on sample size recommendations for a parametric analysis of emBODY data (Nummenmaa et al., 2014), we aimed to recruit 80 participants: 40 individuals with fibromyalgia and 40 pain-free controls. Inclusion criteria were the following: diagnosis of fibromyalgia *or* no history of any persistent pain conditions (pain lasting for ≥ 3 consecutive months), minimum 18 years old, no intellectual disabilities or cognitive impairment, being medication-free or having consistent medication (stable for at least the past month), and fluency in English. As part of a larger project, an additional inclusion criterion was being free of any health condition that prevents safe participation in physical activity (e.g., severe heart or lung disease, uncontrolled diabetes). Participants were recruited via social media, web-based advertisements, rheumatologists who treat fibromyalgia locally, and our existing participant databases.

Participants received a $40 voucher for their time and gained an additional $10 voucher at the study completion as an extra reward for the decision-making part of the project (described elsewhere (Herman et al., 2025).

### Group characteristics

Participants first completed an online screening survey (Qualtrics, Provo, UT) assessing demographics (sex, age, education), medical history, fibromyalgia symptoms (using the **Patient Self-report Survey for the Assessment of Fibromyalgia**, which provides widespread pain index, WPI, and symptom severity scale, SSS (Clauw, 2014), and smoking status. Socioeconomic status was rated using the **MacArthur 10-step self-anchoring scale** (Adler and Stewart 2007).

During the testing session, participants completed additional measures. The **Central Sensitization Inventory (CSI)** Part A (Mayer et al., 2012) assessed symptoms linked to central sensitivity (0–100 scale; α = 0.88, *r* = .82). The **Pain Vigilance and Awareness Questionnaire (PVAQ)** (McCracken, 1997) measured attention to pain (16 items; α = 0.86, *r* = .80). Current pain intensity was rated using a **Visual Rating Scale** (Revill et al., 1976) from 0 (“no pain”) to 10 (“worst pain”). The **Positive and Negative Affect Schedule (PANAS)** (Watson et al. 1988) assessed current mood (α ≥ 0.84 (Crawford and Henry, 2004). The **Depression Anxiety Stress Scale-21 (DASS)** (Lovibond and Lovibond, 1996) measured negative emotional states (α ≥ 0.87 (Antony et al., n.d.).

During testing, participants also completed decision-making tasks (described elsewhere (Herman et al., 2025).

#### Primary outcome: body sensation mapping (the emBODY task)

Using the emBODY tool (Nummenmaa et al., 2014, 2018) (https://version.aalto.fi/gitlab/eglerean/embody*)*, participants indicated increased (termed ‘activations’) or decreased (termed ‘deactivations’) bodily sensations in response to 13 emotional and physiological states (e.g., fear, anger, fatigue, current pain). They coloured human silhouettes accordingly. Instructions emphasised there were no correct answers, and participants could leave items blank if not applicable.

#### Secondary outcomes: bodily sensation perception, interpretation, and alexithymia

Interoceptive beliefs were measured using the **Body Perception Questionnaire-Short Form** (BPQ) (Cabrera et al., 2018). Participants rated awareness of 26 bodily sensations on a 5-point scale; higher scores reflect greater bodily sensation perception (Cronbach’s α > 0.90).

The **Brief Body Sensation Interpretation Questionnaire (BSIQ)** (Clark et al., 1997) assessed interpretation of bodily sensations. It contains 14 ambiguous scenarios (7 internal, 7 external), each with one negative and two neutral/positive explanations. For each scenario, participants rank-ordered these explanations; responses were reverse-scored to reflect negative interpretation tendencies. Internal consistency is acceptable (α = 0.86 for internal, α = 0.74 for external).

These measures were selected due to their relevance to sensations commonly experienced during physical activity (e.g., increased heart rate, sweating); which is recommended by all treatment practice guidelines for Fibromyalgia.

The **Toronto Alexithymia Scale (TAS)** (Bagby et al., 1994) assessed difficulty identifying (DIF)/describing feelings (DDF) and externally oriented thinking style (EOT) with three subscales (α acceptable across psychiatric and non-clinical populations (Preece et al., 2018).

### Data analyses

#### Primary outcome analysis: the emBODY task

The attained sample size (see results) did not allow for parametric analyses (Nummenmaa et al., 2014), but was sufficient for the use of the nonparametric approach to analyse BSMs (Torregrossa et al., 2019). These analyses were performed in Matlab R2020b (The MathWorks, Inc., 2024) and JASP 0.16.1 (JASP Team, 2022).

We reconstructed 50,364-pixel BSMs for each participant from web-based data, coding activations and deactivations as positive and negative values, respectively. Responses outside the body outline were masked and maps were smoothed using a Gaussian kernel (σ = 5). Participants who completed fewer than 50% (i.e., seven) BSMs were excluded (*N* = 1), and all maps were visually screened for anomalies (none removed).

To generate group maps, we calculated per-pixel proportions of positive (P) and negative (N) responses for each body map across participants, resulting in two 50,364 × 13 matrices. From these, we computed a final matrix F = (P + N) × (P − N), capturing both direction (activation vs. deactivation) and consistency of sensations. F values ranged from − 1 to 1, and the colour bar was fixed to this range for both groups to aid visual comparison.

#### Body coverage

Analyses were based on the 12 BSMs shared across groups (excluding the BSM of “your pain today” as not relevant for pain-free controls). We quantified body coverage by comparing the proportion of painted pixels (PPP) using repeated-measures ANOVA (rmANOVA), applying Greenhouse-Geisser correction when necessary.

#### Classification analysis

To classify bodily sensation maps associated with different emotional categories, we implemented a multivariate pattern classification pipeline using linear discriminant analysis (LDA) in MATLAB. Analyses were conducted separately for the fibromyalgia (FM) and control (CO) groups.

Classification performance was evaluated using stratified 5-fold cross-validation. In each fold, the dataset was partitioned into training (80%) and testing (20%) subsets while preserving the proportion of emotion categories across folds. All preprocessing steps were performed independently within each training fold to avoid information leakage.

First, pixel values were standardised (z-scored) using the mean and standard deviation estimated from the training data only. The same transformation parameters were then applied to the corresponding test data. Dimensionality reduction was subsequently performed using principal component analysis (PCA) fitted exclusively on the training data within each fold. To reduce model complexity and minimise overfitting risk in the context of a relatively small sample size, only the first five principal components were retained for classification. These components explained approximately 40–43% of the variance in the data across groups. The trained PCA transformation was then applied to the test data.

The reduced feature representations were classified using linear discriminant analysis (LDA). Predictions from all folds were combined to compute overall classification accuracy and confusion matrices.

To assess whether observed classification accuracy exceeded chance levels, permutation testing was performed separately for each group. Emotion labels were randomly shuffled and the entire classification pipeline, including preprocessing, PCA, and LDA, was repeated 1000 times to generate empirical null distributions of classification accuracy. Observed accuracies were compared against these null distributions to derive permutation-based significance thresholds and p-values.

Cross-validation partitions were generated randomly for each execution of the classification pipeline. Consequently, classification accuracy estimates may differ slightly across repeated runs, including between the primary classification analyses and the permutation-testing procedure, despite identical preprocessing and modelling steps.

The analyses were performed in MATLAB R2024b.

#### Secondary outcomes analysis

Independent samples t-tests, the Welsh test (in case of unequal variances), or *X*^*2*^ (as appropriate) were used to examine group differences in demographic variables, and in bodily awareness and interpretation (BPQ, BSIQ) and alexithymia (TAS). When there were significant group differences in any of the key demographic variables, they were controlled for using analyses of covariance (ANCOVA) when investigating variables of interest (BPQ, BSIQ, TAS). These data did not undergo any transformations. The analyses were performed in JASP 0.16.1.

## Results

### Participants characteristics

Of 157 individuals who completed the pre-screening form, 60 were identified as potentially eligible participants with fibromyalgia. After follow-up, 22 could not be reached and 11 were excluded due to ineligibility (e.g. lacking medical clearance, incorrect diagnosis) or inability to attend in-person testing (residing outside South Australia). Of the 27 invited, five withdrew or missed their appointment. Among the 22 who attended, one withdrew mid-session, one was excluded due to distractibility, and one had incomplete data due to technical issues, resulting in a final sample of 19 people with fibromyalgia. Among 42 eligible pain-free controls, three could not be reached and 18 were excluded due to age or sex mismatch with the fibromyalgia group. Of the 21 invited, one did not attend, and one withdrew. An additional participant was excluded for completing fewer than 50% of the body maps, yielding a final control sample of 18.

### Demographics

Participants had received a fibromyalgia diagnosis an average of 11.84 ± 10.55 years prior (range: 0.5–35 years), though symptom onset was reported 20.11 ± 13.49 years earlier (range: 3–40 years). The median WPI score was 11/19 (range: 6–19), and median SSS score was 4/12 (range: 2–8).

Groups did not differ significantly in age or gender distribution (see Table 1 for details). However, there were significant group differences in SES (fibromyalgia < controls) and years of formal education completed (fibromyalgia < controls). Additionally, as expected, the fibromyalgia group had higher pain ratings, CSI, DASS, and negative affect scores (but not positive affect).

Information about medication used can be found in Supplementary Table 1.

**Table 1:** Group characteristics and comparison. Statistically significant group differences are presented **in bold** (α < 0.05)

Variable		Control			Fibromyalgia			Test statistics	df	*p*	test	Cohen’s d/η²
		*N*	Mean	SD	*N*	Mean	SD					
Demographics	Sex [Male/Female]	3/15			1/18			1.25	1	0.264	X²	
	Age	18	57.61	13.42	19	56.32	13.23	0.30	35	0.769	T	0.10
	Education [years]	18	16.89	2.99	19	14.11	4.32	2.27	35	**0.030**	T	0.75
	SES	18	7.11	1.02	19	5.68	1.64	3.16	35	**0.003**	T	1.04
Pain	Pain Rating	18	0.44	0.78	19	4.47	1.68	-9.43	25.79	**< 0.001**	W	-3.08
	CSI	18	18.67	11.62	19	68.84	10.22	-13.97	35	**< 0.001**	T	-4.59
	PVAQ	18	25.72	12.63	19	56.11	10.40	-8.01	35	**< 0.001**	T	-2.63
PANAS	Positive Affect	18	32.61	6.88	19	29.90	6.69	1.22	35	0.232	T	0.40
	Negative Affect	18	10.78	1.59	19	19.47	9.77	-3.83	19.01	**0.001**	W	-1.24
Depression, Anxiety, Stress Scale (DASS)	Depression	18	1.00	1.61	19	7.26	5.06	-5.13	21.78	**< 0.001**	W	-1.67
	Anxiety	18	0.83	1.58	19	7.00	4.10	-6.10	23.49	**< 0.001**	W	-1.99
	Stress	18	1.94	2.65	19	9.90	4.41	-6.69	29.73	**< 0.001**	W	-2.19
Body Perception Questionnaire (BPQ)		18	42.50	20.90	19	89.26	16.26	32.50	1, 33	**< 0.001**	F	0.48
BSIQ	BSIQ Body Negative	18	1.15	0.23	19	1.55	0.55	5.19	1, 33	**0.029**	F	0.13
	BSIQ Other Negative	18	1.07	0.13	19	1.38	0.48	2.11	1, 33	0.156	F	0.05
Toronto Alexithymia Scale (TAS)	TAS DDF	18	10.39	3.97	19	13.63	5.21	2.24	1, 33	0.144	F	0.06
	TAS DIF	18	11.17	6.31	19	23.47	6.38	23.89	1, 33	**< 0.001**	F	0.42
	TAS EOT	18	17.94	4.12	19	19.68	4.97	0.16	1, 33	0.693	F	0.00

T - independent samples, *t *test, *W* Welsh test, F - ANCOVA

*SES* socioeconomic status, *CSI* Central Sensitisation Index, *PVAQ* Pain Vigilance and Awareness Questionnaire, *PANAS* positive affect/negative affect scale, *BSIQ* Body Sensations Interpretation Questionnaire, *DDF* Difficulty Describing Feelings, *DIF* Difficulty Identifying Feelings, *EOT* Externally Oriented Thinking

## Primary outcome: the emBODY

**Fig. 1:**
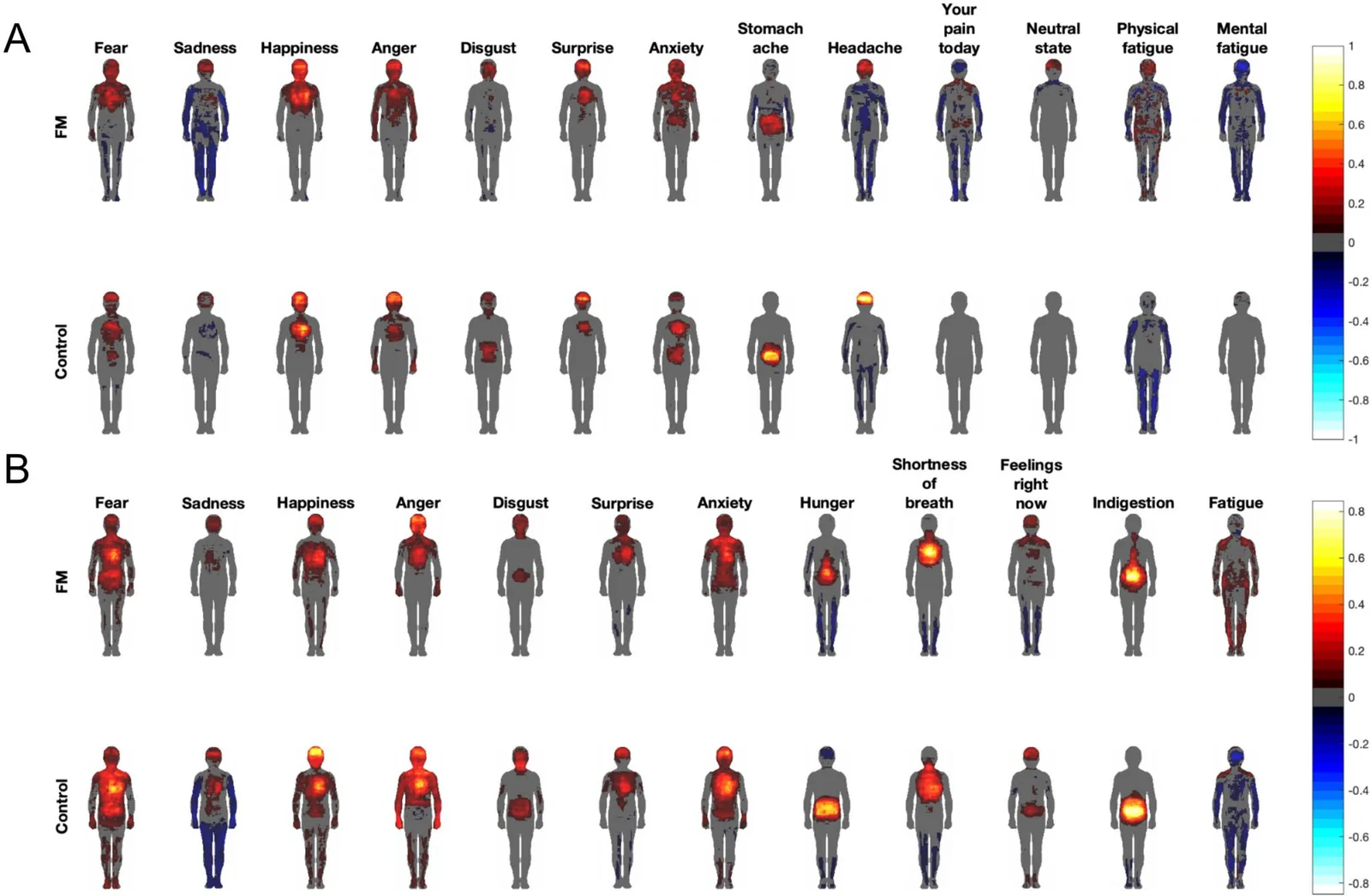
Bodily sensation maps for both groups for study 1 (**A**) and study 2 (**B**) show distributions of body areas where activity was reported to change. The colour bar indicates the proportion of endorsement at each pixel. Warm colours denote activations, and cold colours denote deactivations

On average, participants completed 12.11 ± 0.93 BSMs (minimum = 10; Fig. 1). The maximum pixel endorsement was 1.0 in the control group and 0.54 in the fibromyalgia group, indicating greater variability in the latter group’s responses. Controls showed more localised and distinct bodily maps, while the fibromyalgia group exhibited more widespread and overlapping patterns, particularly in the head and chest, across various states.

### **Body coverage**

**Fig. 2:**
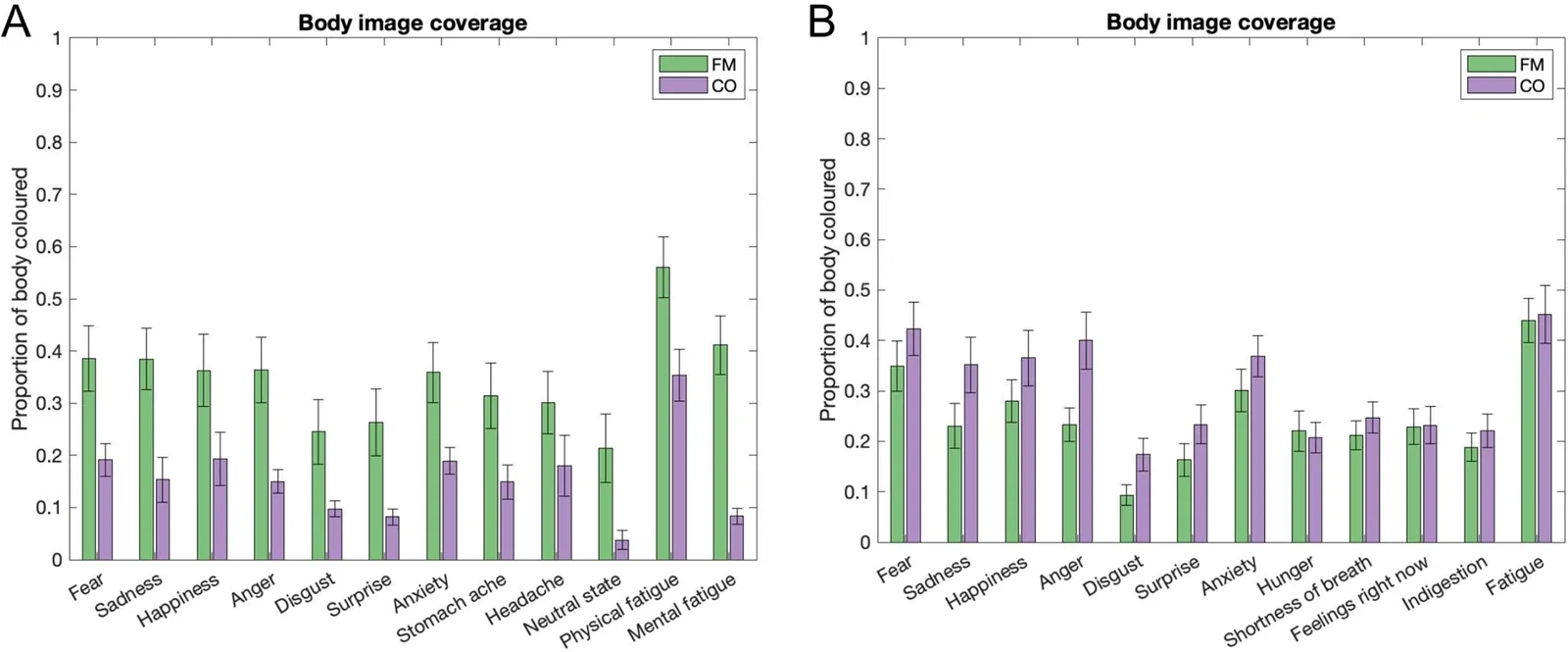
Body coverage for study 1 (**A**) and study 2 (**B**). Bar plot illustrating the proportion of body area coverage for each BSM by group. Error bars indicate the standard error of the mean

The comparison of the proportion of pixels painted revealed the main effect of group, with fibromyalgia group (M = 0.35, SE = 0.04) colouring significantly more pixels than controls [M = 0.16, SE = 0.04; F(1, 35) = 12.00, p = .001, η2 = 0.15], and the main effect of state [Greenhouse-Geisser correction applied: F(6.63, 232.13) = 11.82, p < .001, η2 = 0.10; for posthoc tests see Supplementary Table S2], but no interaction [Greenhouse-Geisser correction applied: F(6.63, 232.13) = 1.18, p = .315, η2 = 0.01; Fig. 2A]. This suggests that people with fibromyalgia perceive specific emotional and non-emotional states over more bodily regions, independent of whether this sensation was painful or not, or whether it is an emotion or other physiological state.

### **Classification accuracy**

Classification accuracy in the fibromyalgia group was 11.8% and 10.2% in the control group, both exceeding the theoretical chance level of 8.3% for 12 categories (t = -338.93, df = 18, p < .001; CO: t = -379.25, df = 17, p < .001; full confusion matrices are depicted in Supplementary Fig. 1). However, permutation testing demonstrated that observed accuracies did not exceed empirically derived chance thresholds in either group (FM = 12.3% p = .264, 95% threshold = 13.6%; CO = 14.8% p = .311, 95% threshold = 16.7%). Thus, classification performance was not reliably above chance when accounting for the high-dimensional structure of the data and the full analysis pipeline.

Because education level and socioeconomic status differed between groups and may plausibly influence task performance, these variables were considered in secondary covariate-adjusted permutation-based analysis. SES and years of education were z-scored and included as covariates. Group differences were tested on covariate-adjusted accuracy values using permutation testing with random reassignment of group labels across participants. BSM-category effects and group × category interactions were evaluated using analogous permutation procedures based on within-subject label shuffling and group relabelling, respectively. All tests were two-tailed and based on 1,000 permutations. To further examine whether classification performance varied across emotion categories and between groups, we conducted a permutation-based covariate-adjusted analysis with BSM category as a within-subject factor and group as a between-subject factor. SES and years of education were included as covariates by residualising accuracy measures prior to inference.

Permutation testing revealed no significant main effect of group, no significant effect of BSM category, and no significant group-by-category interaction (all *p* > .11). These results indicate that classification performance did not differ systematically between fibromyalgia and control participants across BSM categories after accounting for SES and education.

## Secondary outcomes

ANCOVAs, controlling for SES and education, revealed that, compared with controls, the fibromyalgia group had significantly higher scores in BPQ, BSIQ body-related items, and TAS DIF (see Table 1 for details). These findings suggest heightened perception of bodily signals in daily life, more negative interpretation of ambiguous body (but not other) sensations and greater difficulty identifying feelings.

## Study 2

### Methods

We conducted a cross-sectional study involving one in-person testing session at the Laboratory of Brain Imaging undertaken between September 2024 and June 2025. All procedures were approved by the SWPS University Ethics Committee (Ethics Protocol number 238).

#### Participants

This study was a part of a larger neuroimaging project on decision-making in fibromyalgia. We aimed to test 25 participants with fibromyalgia and 25 age, sex, and education-matched pain-free controls. Sample size was estimated based on similar studies utilizing between-group comparisons in decision-making tasks in functional magnetic resonance imaging (fMRI). Inclusion criteria were the following: diagnosis of fibromyalgia *or* no history of any persistent pain conditions (pain lasting for ≥ 3 consecutive months), 18 to 55 years old, no intellectual disabilities or cognitive impairment, being medication-free or having consistent medication (stable for at least the past month), and normal or corrected-to-normal vision. As part of a functional magnetic resonance imaging (fMRI) study, participants also had to meet criteria for safe participation in the fMRI study (i.e. no unremovable metal elements in the body, no pregnancy, and BMI ≤ 30). Participants were recruited via social media, web-based advertisements, and local associations of individuals with FM.

Participants received PLN200 for their participation.

#### Group characteristics

Participants first completed an online screening administered through Limesurvey (LimeSurvey GmbH, n.d.), assessing demographics (sex, age, education, height and weight, information about FM diagnosis) and fMRI safety questions.

During the testing session, participants completed additional measures assessing medical history, and fibromyalgia symptoms (when applicable, using the **Patient Self-report Survey for the Assessment of Fibromyalgia** (Häuser et al., 2017; Wolfe et al., 2016). The Polish version of the **Central Sensitization Inventory (CSI)** Part A and B (Turczyn et al., 2019; Neblett, 2018) assessed symptoms linked to central sensitivity (0–100 scale; α = 0.88, *r* = .82). Current pain intensity was rated using a **Visual Rating Scale** (Revill et al., 1976) from 0 (“no pain”) to 10 (“worst pain”). The Polish version of the **Positive and Negative Affect Schedule (PANAS)** (Wróbel et al., 2019; Laurent et al., 1999) assessed current mood (α ≥ 0.84 (Wróbel et al., 2019). The Polish version of the **Depression Anxiety Stress Scale-21 (DASS)** (Lovibond and Lovibond, 1996; Makara-Studzińska et al., 2024) measured negative emotional states (α ≥ 0.89 (Makara-Studzińska et al., 2024).

## Primary outcome: body sensation mapping (the emBODY task)

Using the emBODY tool (Nummenmaa et al., 2014, 2018) (https://version.aalto.fi/gitlab/eglerean/embody*)*, participants indicated increased (termed ‘activations’) or decreased (termed ‘deactivations’) bodily sensations in response to 12 emotional and physiological states (e.g., fear, anger, fatigue, hunger). They coloured human silhouettes accordingly. Instructions emphasised there were no correct answers, and participants could leave items blank if not applicable.

## Secondary outcomes: interoception and alexithymia

**The Polish version of the Perth Alexithymia Questionnaire-Short Form (PAQ-SF)** (Larionow et al., 2023; Preece et al., 2023) has a brief, 6-item format designed to enable alexithymia assessments in more time-pressured settings. All PAQ-SF items are answered on a 7-point Likert scale, with higher scores indicating higher alexithymia. The Polish version of the scale shows a good internal consistency (Cronbach’s α = 0.81).

The **Interoception Sensory Questionnaire (ISQ-8)** (Suzman et al., 2021) is an eight-item version of the previously proposed longer version, ISQ-20 (Fiene et al., 2018). It was developed to assess interoceptive challenges (e.g., ‘I have difficulty making sense of my body’s signals unless they are very strong’ or ‘I find it difficult to describe feelings like hunger, thirst, hot or cold’). Items are rated on a five-point scale (1–5) with higher scores indicating greater interoceptive difficulties. The Cronbach’s α of the ISQ is high (0.96) (Suzman et al., 2021).

The **Heartbeat Tracking (HBT) Task** (Garfinkel et al., 2015; Schandry, 1981) assesses interoceptive accuracy (IAcc) (Garfinkel et al., 2015). Participants were instructed to count, without manually checking, heartbeats they felt in the body during variable periods. These ratings were compared against the actual number of heartbeats, as recorded objectively and noninvasively by a clinical-grade pulse oximeter (Nonin Inc.) fitted with a soft cuff, placed over the participant’s index or middle finger of their non-dominant hand. Six trials with variable time windows of 25, 30, 35, 40, 45, and 50 s were presented in a randomised order. The IAcc score equalled 1/6 ∑ [1 − (|nbeats real − nbeats reported|)/((nbeats real + nbeats reported)/2)] (Hart et al., 2013).

Notably, performance in the HBT task can potentially be affected by various factors, including knowledge of one’s heart rate or counting seconds instead of heartbeats; thus, its validity has recently been criticised (e.g., (Ring and Brener, 2018; Desmedt et al., 2020) but see (Schulz et al., 2021; Ainley et al., 2020) for further discussion). According to recent recommendations (Desmedt et al., 2020), we provided clear instructions, explicitly stating that participants should not guess or try to estimate their heart rate in any way, only report heartbeats that they actually feel, and assured participants that reporting no beats at all was acceptable as well.

At the end of each trial, participants immediately rated how confident they were in their answers on a scale of 0-100, where 0 denoted a guess and 100 denoted complete confidence.

We also computed **interoceptive insight** scores (the metacognitive awareness of one’s performance), which reflect the extent to which confidence predicts task accuracy (Garfinkel et al., 2015; Khalsa et al., 2018). The insight score for the HBT task was assessed by calculating the within-participant Pearson correlation, *r*, between confidence and accuracy scores.

During testing, participants also completed decision-making tasks in the fMRI scanner, which will be reported elsewhere.

### Data analyses

The analyses were conducted analogously to Study 1.

## Results

### Group characteristics

26 (3 men) individuals with a diagnosis of FM and 25 (2 men) individuals with no history of persistent pain (control group) participated in the study. One male participant was excluded from the analysis due to marked distractibility during the experimental session and subsequent attempts to re-enrol in the study using different personal information. Additionally, one male participant in the control group completed fewer than 50% of the body maps and was therefore removed from all analyses, resulting in a final sample of 49 participants. Participants had received a fibromyalgia diagnosis on average 3.4 ± 2.85 years prior (range: 0.5–10 years, median 2). The median WPI score was 13/19 (range: 2–18), and the median SSS score was 6/12 (range: 5–9).

The summary of demographic information is presented in Table 2. Groups did not differ in sex distribution, age or education level, however, as expected, FM group presented significantly higher level of pain ratings, higher index of central sensitization (FM group at the the extremely severe level, while the control group at the subclinical level (Neblett et al., 2017). FM group also showed higher scores on all DASS subscales together with higher reports of negative affect and lower reports of positive affect. Information about medication used can be found in Supplementary Table 3.

**Table 2:** Study 2 group characteristics and comparison. **In bold** are presented statistically significant group differences (α < 0.05)

							95% CI for mean difference			Control			FM		
	t	df	*p*		Mean difference	SE difference	Lower	Upper	Cohen’s d	Mean/*N*	SD	SE	Mean/*N*	SD	SE
Age	-0.34	48.00	0.738		-0.88	2.62	-6.14	4.38	-0.10	30.88	8.89	1.78	31.76	9.60	1.92
DASS D	-5.09	38.27	**< 0.001**	**ᵃ**	-6.24	1.23	-8.70	-3.78	-1.44	2.68	3.05	0.61	8.92	5.31	1.06
DASS A	-6.03	33.09	**< 0.001**	**ᵃ**	-6.12	1.01	-8.16	-4.08	-1.71	1.68	2.06	0.41	7.80	4.64	0.93
DASS S	-5.67	48.00	**< 0.001**		-6.28	1.11	-8.51	-4.05	-1.60	4.44	4.09	0.82	10.72	3.74	0.75
PANAS NA	-4.90	37.64	**< 0.001**	**ᵃ**	-5.16	1.05	-7.28	-3.04	-1.39	8.48	2.57	0.51	13.64	4.60	0.92
PANAS PA	4.07	48.00	**< 0.001**		4.84	1.19	2.45	7.23	1.15	16.52	4.71	0.94	11.68	3.63	0.73
PAQ	-2.31	48.00	**0.026**		-5.32	2.31	-9.96	-0.68	-0.65	15.12	7.09	1.42	20.44	9.11	1.82
CSI A	-10.16	48.00	**< 0.001**		-36.52	3.60	-43.75	-29.29	-2.87	28.64	13.68	2.74	65.16	11.66	2.33
CSI B	-6.91	38.41	**< 0.001**	**ᵃ**	-3.16	0.46	-4.08	-2.24	-1.96	0.84	1.14	0.23	4.00	1.98	0.40
ISQ	-4.55	48.00	**< 0.001**		-7.48	1.64	-10.78	-4.18	-1.29	11.96	5.07	1.01	19.44	6.46	1.29
IAcc	1.34	48.00	0.187		0.21	0.16	-0.11	0.53	0.38	0.23	0.56	0.11	0.01	0.56	0.11
IConfidence	1.47	37.17	0.150	**ᵃ**	0.98	0.66	-0.36	2.31	0.42	5.89	1.59	0.32	4.92	2.91	0.58
IInsight	-1.72	43.00	0.092		-0.24	0.14	-0.52	0.04	-0.52	0.30	0.51	0.10	0.54	0.42	0.09
HR	-1.39	48.00	0.173		-4.62	3.33	-11.32	2.09	-0.39	74.98	11.11	2.22	79.60	12.42	2.49
Education	0.64	2	0.725	**b**											

ᵃWelsh test, ^b^ Χ^2^

*CSI* Central Sensitisation Index, *PANAS* positive affect/negative affect scale

### Primary outcomes

On average, participants completed 11.43 ± 1 BSMs (minimum = 8; Fig. 1B). The maximum pixel endorsement was 0.84 in the control group and 0.85 in the fibromyalgia group, indicating similar response variability in both groups.

#### **Body coverage**

The comparison of the proportion of pixels painted revealed the main effect of BSM [Greenhouse-Geisser correction applied: F(7.46, 350.68) = 16.62, p < .001, η2 = 0.15; for posthoc tests see Supplementary Table S4], but no main effect of group [F(1, 47) = 2.32, p = .135, η2 = 0.02] or interaction [Greenhouse-Geisser correction applied: F(7.46, 350.68) = 1.36, p = .216, η2 = 0.01; Fig. 2B]; therefore indicating that participants in both groups coloured in comparable body surface.

#### **Classification accuracy**

Classification accuracy in the fibromyalgia group was 16.3% and 17.4% in the control group, both exceeding the theoretical chance level of 8.3% for 12 categories (FM: t = -503.14, df = 24, p < .001; CO: t = 4.14, df = 23, p < .001; full confusion matrices are depicted in Supplementary Fig. 2). However, permutation testing demonstrated that observed accuracies did not exceed empirically derived chance thresholds in either group (FM = 18.3%, p = .092, 95% threshold = 18.7%; CO = 18.1%, p = .468, 95% threshold = 20.5%). Thus, classification performance was not reliably above chance when accounting for the high-dimensional structure of the data and the full analysis pipeline.

No significant difference in classification performance was observed between fibromyalgia and control participants (*p* = .701).

### Secondary outcomes

Compared to the control group, the FM group reported higher levels of alexithymia (as assessed with the PAQ). There were no significant group differences in interoceptive accuracy, insight or confidence; however, the FM group reported a higher level of interoceptive difficulties (ISQ) than the control group. See Table 2 for details.

## Discussion

This research aimed to explore the perception, interpretation, and embodiment of bodily signals in individuals with fibromyalgia compared to pain-free controls. Across two studies, recruiting independent samples, we observed marked group differences across most domains. Fibromyalgia participants showed heightened perception of bodily sensations (BPQ, Study 1), more negative interpretation of ambiguous body-related cues (BSIQ, Study 1), greater interoceptive difficulty (ISQ, Study 2), and heightened alexithymia levels (TAS-DIF, PAQ, Studies 1 and 2) than controls. Yet, we found no group differences in interoceptive accuracy or insight (Study 2) or differences in bodily sensations differentiation. Together, these findings suggest that fibromyalgia is associated with altered interoceptive beliefs and attributions, but not necessarily emotion–body integration. Overall, our findings reinforce the connection between pain and emotional processing circuits and support the view that nociplastic pain conditions, like fibromyalgia, may disrupt the somatovisceral experience of emotion, holding relevant theoretical and clinical implications.

Participants mapped emotions onto similar bodily regions, consistent with prior research (Ojala et al., 2023; Nummenmaa et al., 2014, 2018; Volynets et al., 2020; Herman et al., 2024). One exception is disgust, where controls reported sensations in the head and abdomen, and those with fibromyalgia reported sensations in the head only. In Study 2, sadness and fatigue were also more related to deactivations throughout the body in the control group compared to the FM group. This suggests that the topography of bodily sensations remains largely intact in fibromyalgia. Yet, those with fibromyalgia in Study 1 reported more widespread bodily experiences for a range of states, including not only pain but also emotional and fatigue-related sensations. No such differences were observed in Study 2. However, differences in group characteristics between the two studies may be relevant. Notably, participants in Study 1 were older (M = 56.95 ± 12.15, range 26–79) than participants in Study 2 (M = 31.32 ± 9.17, range 20–53); therefore, participants in Study 1 had longer disease onset and, presumably, treatment, resulting in some differences in symptom severity, which may have also affected the perception of bodily sensations in the emBODY task. Future studies should investigate this aspect in a larger sample. Interestingly, previous research in chronic pain samples (Ojala et al., 2023), such as those with complex regional pain syndrome or neuropathic pain, reported reduced embodiment of emotions. Thus, our findings may also be related to unique features of nociplastic pain conditions like fibromyalgia, which are associated with central nervous system sensitisation and distributed pain patterns.

We hypothesised that individuals with fibromyalgia would exhibit a diminished ability to differentiate between bodily states, yet the rigorous and conservative linear discriminant analysis (LDA) approach used suggested no such differences across BSMs in both studies. Of interest, our initial analyses following published pipelines *(*Nummenmaa et al., 2014; Lyons et al., 2021) using a less rigorous LDA approach suggested group differences in bodily-state differentiation, but these differences were not replicated when more stringent analytical procedures were applied. This pattern highlights the sensitivity of LDA findings to analytic choices and suggests that any potential group differences should be interpreted cautiously until replicated using robust methodologies.

Current results go against previous work using rating tasks, which found less distinct perception of emotional and physiological states in individuals with chronic pain (Lin et al., 2024). These discrepancies might be due to a rather modest sample size in the current studies. Indeed, previous works using the emBODY tool usually employed greater samples (Herman et al., 2024; Nummenmaa et al., 2014; Hietanen et al., 2016). Yet, questionnaire findings showed that participants with fibromyalgia reported greater subjective interoceptive difficulties (ISQ) that can be interpreted as representing confusion about interoceptive bodily states unless these states are extreme (Alexisomia) (Fiene et al., 2018). Participants with FM also showed higher perception of bodily sensations (indexed by BPQ) likely to occur during anxiety or fatigue. They also reported greater difficulty in describing and identifying emotions (TAS, PAQ) and demonstrated a negative interpretation bias toward ambiguous bodily sensations (BSIQ). Together, these findings point to subjective interoceptive difficulties and altered identification and interpretation of internal signals in fibromyalgia, consistent with literature reporting heightened interoceptive sensibility, alexithymia, and impaired emotion recognition in this condition (Ciuffini et al., 2023; Muñoz Ladrón de Guevara et al., 2021; Montoro et al., 2016; Rost et al., 2017).

Importantly, performance on the objective cardiac perception task (HBT) did not differ between groups, nor did confidence ratings or interoceptive insight. This pattern suggests either that fibromyalgia is characterised by specific alterations in interoceptive beliefs and bodily attributions rather than in interoceptive accuracy per se, or that other interoceptive modalities (e.g., respiratory or gastric) are more strongly affected than cardiac interoception. Previous findings on cardiac interoception in fibromyalgia are mixed: some studies report no group differences in interoceptive accuracy (Rost et al., 2017; Borg et al., 2018), others report reduced accuracy in fibromyalgia (Duschek et al., 2017), while a more recent study using an alternative paradigm suggests heightened interoceptive sensitivity, operationalised as superior adjustment of exteroceptive stimuli to one’s heartbeat, in individuals with fibromyalgia compared with controls (Todd et al., 2024). Together, these inconsistencies highlight the need for future studies to assess interoceptive accuracy across multiple interoceptive modalities using converging methodological approaches.

Overall, our results support recent proposals that interoceptive signals may be noisier in chronic pain (Lin et al., 2024), increasing reliance on top-down predictions (e.g., beliefs and expectations). In Study 1, individuals with fibromyalgia interpreted ambiguous bodily sensations more negatively than controls, while showing no difference in interpretation of general events, suggesting a specific interoceptive misattribution rather than a general negative cognitive style. Additionally, in Study 2, we found that individuals with FM reported greater subjective challenges in detecting bodily sensations. If sensory signals are ambiguous or imprecise, individuals may default to prior beliefs, potentially leading to bodily experiences that align with negative expectations (Moseley and Vlaeyen, 2015; Meulders et al., 2014). These findings offer insight into how negative emotion and somatic distress may be conceptualised and perpetuated in fibromyalgia. Thus, our results suggest that the primary difficulty in FM may lie not in perceiving bodily sensations, but in conceptualising, interpreting, and attributing meaning to those sensations. This interpretation is consistent with the concept of alexisomia, which describes difficulties in recognising and making sense of bodily experiences despite the presence of bodily sensations themselves (Oka 2020). Such a distinction may help explain why participants with fibromyalgia reported greater interoceptive difficulties while showing comparable performance on the body mapping task and HBT.

These findings raise an important clinical question: Could training focused on bodily sensation interpretation improve clinical symptom and reduce pain-related distress in fibromyalgia? Notably, the absence of group differences in interoceptive accuracy in the present study suggests that targeting accuracy per se may not be a supported therapeutic target in fibromyalgia. However, our results point to altered bodily interpretation and attribution processes, characterised by more negative interpretations of ambiguous bodily cues.

Interventions targeting emotional awareness, such as Emotional Awareness and Expression Therapy, are already available for chronic pain (Lumley and Schubiner, 2019) and have been tested against cognitive behavioural therapy and pain education for people with fibromyalgia with promising results (Lumley et al., 2017). The therapy encourages patients to reinterpret symptoms as manifestations of emotional nervous system processes and to process and express emotions associated with past adversity, trauma, or interpersonal conflict. Eight-session intervention was well received, more effective than a basic educational intervention, and had advantages over cognitive behavioural therapy on pain. Our results suggest that targeting emotional experience and interpretation of bodily signals might bring particular benefit in FM.

### Limitations

Some limitations should be considered when interpreting the present findings. First, recruitment proved challenging and, due to time and funding constraints, Study 1 was conducted with a smaller sample size than originally planned (target: 40 participants per group). Given the relatively high dimensionality of the bodily map data in relation to sample size, the multivariate classification analyses should be interpreted cautiously despite the use of dimensionality reduction, cross-validation, and permutation-based robustness analyses. Future studies with substantially larger samples will be important for establishing the stability and generalisability of these findings.

Second, one of the main visual findings from Study 1, greater body coverage in the FM group relative to controls, was not replicated in Study 2. Differences in age and disease duration between samples may contribute to this discrepancy. Somatic awareness, emotional processing, and interoceptive sensibility are known to change across the lifespan (Carr et al., 2024; Mikkelsen et al., 2019; Hietanen et al., 2016), making it difficult to disentangle the effects of age and chronicity in the present design.

Third, Study 2 involved additional selection constraints related to MRI safety procedures, including exclusion of participants with BMI > 30. Given the high prevalence of obesity in fibromyalgia and the known associations between BMI, pain, inflammation, and interoceptive processing (Mathkhor & Ibraheem, 2023; Robinson et al., 2021), this may have reduced the representativeness of the clinical sample and limited generalisability of the findings to the broader fibromyalgia population.

Several additional factors may also have influenced performance on the emBODY task, including medication use, socioeconomic differences, educational background, and variability in symptom chronicity. While these factors are difficult to fully disentangle from the clinical phenotype itself, they may contribute to heterogeneity in emotional and bodily reporting and should be systematically examined in future work.

Importantly, the emBODY paradigm captures the spatial location of bodily sensations but does not assess their qualitative characteristics. Consequently, overlapping bodily maps do not necessarily imply identical subjective emotional experiences. For example, two emotions may be localised to the same body region while differing substantially in sensory quality or affective interpretation. This distinction may be particularly relevant in fibromyalgia, where altered interpretation of bodily signals and negative interpretative biases have previously been proposed. In this context, the present findings may reflect differences in the interpretation or conceptualisation of bodily sensations rather than reduced bodily detection per se.

## Conclusions and future directions

Our findings reveal increased self-reported interoceptive difficulties alongside more negative interpretations of ambiguous bodily cues in individuals with fibromyalgia. These disruptions in emotion-body integration may contribute to heightened pain and emotional distress, providing a promising target for therapeutic strategies. Interventions such as interoceptive training could help recalibrate the way individuals interpret bodily information. Longitudinal studies are needed to examine the impact of such interventions on pain outcomes, emotional well-being, and daily functioning in fibromyalgia.

## Supplementary Information

Below is the link to the electronic supplementary material.


Supplementary Material 1


## Data Availability

Data and analyses scripts are available via Open Science Framework (OSF): < https://osf.io/4du3f/overview?view_only=9f346447658e465f896b02275ba712f8>.
