# Supplementary material for: When emotions hurt: negative interpretations of bodily signals and interoceptive difficulties in fibromyalgia
Source: Psychol Res. 2026 Jul 2;90(4):125. doi: 10.1007/s00426-026-02341-2 (PMC13328158; doi:10.1007/s00426-026-02341-2)

Supplementary Materials

Table S1

Information about medication for each participant in Study 1. IDs 101 to 118 denote the FM group, 201 to 219 - the control group.

ID	SNRI	SSRI	TCA	Gabapentinoid	Antiepileptic	Opioid
101	0	1	0	0	0	0
102	0	0	0	0	0	0
103	0	1	0	0	0	1
104	0	0	0	0	0	1
105	1	0	0	0	0	0
106	0	1	0	0	0	1
107	1	0	1	0	0	0
108	1	0	0	0	0	0
110	0	1	0	0	0	0
111	1	1	0	0	0	0
112	0	0	0	0	0	1
113	0	0	0	0	0	0
114	1	0	0	1	0	0
115	0	0	0	0	0	1
116	0	0	0	0	0	0
117	0	0	0	0	0	0
118	1	0	0	0	0	0
120	1	0	0	0	0	0
122	0	0	1	0	0	0
201	0	0	0	0	0	0
202	0	0	0	0	0	0
203	0	0	0	0	0	0
204	0	1	0	0	0	0
205	0	0	0	0	0	0
206	0	0	0	0	0	0
207	0	1	0	0	0	0
208	0	0	0	0	0	0
209	0	0	1	0	0	0

211	0	0	0	0	0	0
212	0	0	0	0	0	0
213	0	0	0	0	0	0
214	0	0	0	0	0	0
215	0	0	0	0	0	0
216	0	0	0	0	0	0
217	0	0	0	0	0	0
218	0	0	0	0	0	0
219	0	0	0	0	0	0
**FM sum:**	**7**	**5**	**2**	**1**	**0**	**5**
**CN sum:**	**0**	**2**	**1**	**0**	**0**	**0**

Table S2

Post Hoc Comparisons for the proportions of pixels painted between body sensation maps in Study 1.

		Mean Difference	SE	t	Cohen's d	p_{holm}
Fear	Sadness	0.020	0.034	0.580	0.090	1.000
	Happiness	0.010	0.034	0.299	0.046	1.000
	Anger	0.031	0.034	0.932	0.144	1.000
	Disgust	0.117	0.034	3.464	0.535	**0.029**
	Surprise	0.116	0.034	3.422	0.529	**0.033**
	Anxiety	0.014	0.034	0.424	0.065	1.000
	Stomach ache	0.057	0.034	1.678	0.259	1.000
	Headache	0.048	0.034	1.419	0.219	1.000
	Neutral state	0.163	0.034	4.821	0.745	**< .001**
	Physical fatigue	-0.169	0.034	-4.992	-0.772	**< .001**
	Mental fatigue	0.041	0.034	1.214	0.188	1.000
Sadness	Happiness	-0.009	0.034	-0.281	-0.043	1.000
	Anger	0.012	0.034	0.352	0.054	1.000
	Disgust	0.097	0.034	2.884	0.446	0.170
	Surprise	0.096	0.034	2.842	0.439	0.189
	Anxiety	-0.005	0.034	-0.156	-0.024	1.000
	Stomach ache	0.037	0.034	1.098	0.170	1.000
	Headache	0.028	0.034	0.840	0.130	1.000
	Neutral state	0.143	0.034	4.242	0.656	**0.001**
	Physical fatigue	-0.188	0.034	-5.572	-0.861	**< .001**
	Mental fatigue	0.021	0.034	0.635	0.098	1.000
Happiness	Anger	0.021	0.034	0.633	0.098	1.000
	Disgust	0.107	0.034	3.165	0.489	0.077
	Surprise	0.105	0.034	3.123	0.483	0.085
	Anxiety	0.004	0.034	0.125	0.019	1.000
	Stomach ache	0.047	0.034	1.379	0.213	1.000
	Headache	0.038	0.034	1.120	0.173	1.000
	Neutral state	0.153	0.034	4.522	0.699	**< .001**
	Physical fatigue	-0.179	0.034	-5.291	-0.818	**< .001**
	Mental fatigue	0.031	0.034	0.915	0.141	1.000
Anger	Disgust	0.086	0.034	2.532	0.391	0.458
	Surprise	0.084	0.034	2.490	0.385	0.502
	Anxiety	-0.017	0.034	-0.508	-0.079	1.000
	Stomach ache	0.025	0.034	0.746	0.115	1.000
	Headache	0.016	0.034	0.487	0.075	1.000
	Neutral state	0.131	0.034	3.889	0.601	**0.006**

	Physical fatigue	-0.200	0.034	-5.924	-0.916	**< .001**
	Mental fatigue	0.010	0.034	0.282	0.044	1.000
Disgust	Surprise	-0.001	0.034	-0.042	-0.007	1.000
	Anxiety	-0.103	0.034	-3.040	-0.470	0.109
	Stomach ache	-0.060	0.034	-1.786	-0.276	1.000
	Headache	-0.069	0.034	-2.045	-0.316	1.000
	Neutral state	0.046	0.034	1.357	0.210	1.000
	Physical fatigue	-0.286	0.034	-8.456	-1.307	**< .001**
	Mental fatigue	-0.076	0.034	-2.250	-0.348	0.926
Surprise	Anxiety	-0.101	0.034	-2.998	-0.463	0.122
	Stomach ache	-0.059	0.034	-1.744	-0.270	1.000
	Headache	-0.068	0.034	-2.002	-0.309	1.000
	Neutral state	0.047	0.034	1.400	0.216	1.000
	Physical fatigue	-0.284	0.034	-8.414	-1.300	**< .001**
	Mental fatigue	-0.075	0.034	-2.207	-0.341	1.000
Anxiety	Stomach ache	0.042	0.034	1.254	0.194	1.000
	Headache	0.034	0.034	0.996	0.154	1.000
	Neutral state	0.149	0.034	4.398	0.680	**< .001**
	Physical fatigue	-0.183	0.034	-5.416	-0.837	**< .001**
	Mental fatigue	0.027	0.034	0.791	0.122	1.000
Stomach ache	Headache	-0.009	0.034	-0.258	-0.040	1.000
	Neutral state	0.106	0.034	3.144	0.486	0.081
	Physical fatigue	-0.225	0.034	-6.670	-1.031	**< .001**
	Mental fatigue	-0.016	0.034	-0.463	-0.072	1.000
Headache	Neutral state	0.115	0.034	3.402	0.526	0.035
	Physical fatigue	-0.217	0.034	-6.411	-0.991	**< .001**
	Mental fatigue	-0.007	0.034	-0.205	-0.032	1.000
Neutral state	Physical fatigue	-0.332	0.034	-9.813	-1.517	**< .001**
	Mental fatigue	-0.122	0.034	-3.607	-0.558	**0.018**
Physical fatigue	Mental fatigue	0.210	0.034	6.206	0.959	**< .001**

Note. P-value adjusted for comparing a family of 66

Note. Results are averaged over the levels of: Group

Note. Significant comparisons are depicted in **bold**.

Figure S1

Confusion matrices for classification of bodily sensation maps in fibromyalgia and control groups in Study 1.

Row-normalised confusion matrices are shown for the fibromyalgia group (A) and the control group (B), expressed as percentages of correctly and incorrectly classified trials for each true label. Each row represents the true category and each column represents the predicted category, such that values reflect the proportion of trials from a given category assigned to each predicted label. Diagonal elements indicate correct classifications, while off-diagonal elements reflect misclassifications between categories. The colour scale is matched across panels and normalised to the maximum percentage observed in the control group to ensure comparability. White shading corresponds to performance below the theoretical chance level of 8%.

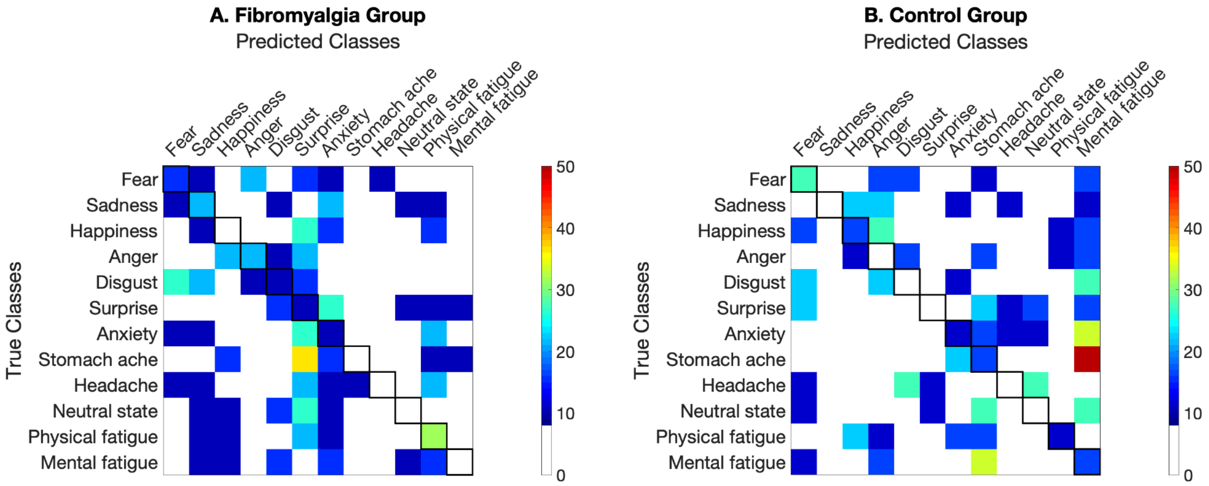

Table S3

Information about medication for each participant in Study 2. IDs 100 to 125 denote the FM group, 200 to 225 - the control group.

ID	SNRI	SSRI	TCA	Gabapentinoid	Antiepileptic	Opioid
100	0	0	0	1	0	0
101	1	0	0	1	0	0
102	0	0	1	0	0	0
103	0	1	0	0	0	0
104	1	0	0	0	0	0
105	1	0	0	1	0	0
107	1	1	0	1	0	0
108	1	0	1	0	0	0
109	1	0	0	1	0	0
110	0	1	0	0	0	0
111	0	0	0	0	0	0
112	1	0	0	0	0	0
113	1	0	0	1	0	0
114	1	0	0	1	1	0
115	1	0	0	1	0	0
116	1	0	0	1	0	0
117	1	0	0	0	0	0
118	1	0	1	1	1	0
119	1	0	0	0	0	0
120	0	0	0	0	0	0
121	0	0	0	0	0	0
122	0	0	1	0	0	0
123	1	0	0	0	0	0
124	1	0	0	1	0	0
125	0	0	0	0	0	0
200	0	0	0	0	0	0
201	1	0	0	0	0	0
202	0	0	0	0	0	0
203	0	0	0	0	0	0
204	0	0	0	0	0	0
205	0	0	0	0	0	0

207	1	1	0	1	0	0
208	0	1	0	0	0	0
209	0	0	0	0	0	0
210	0	0	0	0	0	0
211	0	0	0	0	0	0
212	0	0	0	0	0	0
213	0	0	0	0	0	0
214	0	0	0	0	0	0
216	0	0	0	0	0	0
217	0	0	0	0	0	0
218	0	0	0	0	0	0
219	0	0	0	0	0	0
220	0	0	0	0	0	0
221	0	0	0	0	0	0
222	0	0	0	0	0	0
223	1	1	0	0	0	0
224	0	0	0	0	0	0
225	0	0	0	0	0	0
**FM sum:**	**16**	**3**	**4**	**11**	**2**	**0**
**CN sum:**	**3**	**3**	**0**	**1**	**0**	**0**

Table S4

Post Hoc Comparisons for the proportions of pixels painted between body sensation maps in Study 2.

		Mean Difference	SE	t	p_{holm}
Fear	Sadness	0.095	0.031	3.057	0.078
	Happiness	0.063	0.031	2.038	0.882
	Anger	0.069	0.031	2.241	0.585
	Disgust	0.252	0.031	8.141	**< .001**
	Surprise	0.187	0.031	6.045	**< .001**
	Anxiety	0.051	0.031	1.640	1.000
	Hunger	0.172	0.031	5.547	**< .001**
	Shortness of breath	0.156	0.031	5.044	**< .001**
	Feelings right now	0.155	0.031	5.011	**< .001**
	Indigestion	0.181	0.031	5.838	**< .001**
	Fatigue	-0.060	0.031	-1.928	0.979
Sadness	Happiness	-0.032	0.031	-1.019	1.000
	Anger	-0.025	0.031	-0.816	1.000
	Disgust	0.158	0.031	5.083	**< .001**
	Surprise	0.093	0.031	2.988	0.091
	Anxiety	-0.044	0.031	-1.418	1.000
	Hunger	0.077	0.031	2.490	0.327
	Shortness of breath	0.062	0.031	1.986	0.950
	Feelings right now	0.061	0.031	1.954	0.974
	Indigestion	0.086	0.031	2.781	0.157
	Fatigue	-0.155	0.031	-4.985	**< .001**
Happiness	Anger	0.006	0.031	0.202	1.000
	Disgust	0.189	0.031	6.102	**< .001**
	Surprise	0.124	0.031	4.006	**0.003**
	Anxiety	-0.012	0.031	-0.399	1.000
	Hunger	0.109	0.031	3.509	**0.019**
	Shortness of breath	0.093	0.031	3.005	0.089
	Feelings right now	0.092	0.031	2.973	0.093
	Indigestion	0.118	0.031	3.800	**0.007**
	Fatigue	-0.123	0.031	-3.966	**0.004**
Anger	Disgust	0.183	0.031	5.900	**< .001**
	Surprise	0.118	0.031	3.804	**0.007**
	Anxiety	-0.019	0.031	-0.601	1.000
	Hunger	0.103	0.031	3.306	**0.036**
	Shortness of breath	0.087	0.031	2.803	0.152
	Feelings right now	0.086	0.031	2.770	0.157
	Indigestion	0.112	0.031	3.597	**0.014**
	Fatigue	-0.129	0.031	-4.169	**0.002**
Disgust	Surprise	-0.065	0.031	-2.096	0.805
	Anxiety	-0.202	0.031	-6.501	**< .001**
	Hunger	-0.080	0.031	-2.593	0.254
	Shortness of breath	-0.096	0.031	-3.097	0.070
	Feelings right now	-0.097	0.031	-3.130	0.065
	Indigestion	-0.071	0.031	-2.302	0.521
	Fatigue	-0.312	0.031	-10.069	**< .001**
Surprise	Anxiety	-0.137	0.031	-4.405	**< .001**
	Hunger	-0.015	0.031	-0.498	1.000
	Shortness of breath	-0.031	0.031	-1.001	1.000

	Feelings right now	-0.032	0.031	-1.034	1.000
	Indigestion	-0.006	0.031	-0.207	1.000
	Fatigue	-0.247	0.031	-7.973	**< .001**
Anxiety	Hunger	0.121	0.031	3.907	**0.005**
	Shortness of breath	0.106	0.031	3.404	**0.027**
	Feelings right now	0.105	0.031	3.371	**0.030**
	Indigestion	0.130	0.031	4.198	**0.002**
	Fatigue	-0.111	0.031	-3.568	**0.016**
Hunger	Shortness of breath	-0.016	0.031	-0.503	1.000
	Feelings right now	-0.017	0.031	-0.536	1.000
	Indigestion	0.009	0.031	0.291	1.000
	Fatigue	-0.232	0.031	-7.475	**< .001**
Shortness of breath	Feelings right now	-0.001	0.031	-0.033	1.000
	Indigestion	0.025	0.031	0.794	1.000
	Fatigue	-0.216	0.031	-6.972	**< .001**
Feelings right now	Indigestion	0.026	0.031	0.827	1.000
	Fatigue	-0.215	0.031	-6.939	**< .001**
Indigestion	Fatigue	-0.241	0.031	-7.766	**< .001**

Note. P-value and confidence intervals adjusted for comparing a family of 66 estimates (confidence intervals corrected using the bonferroni method).
Note. Results are averaged over the levels of: Group
Note. Significant comparisons are depicted in **bold**.

Figure S2

Confusion matrices for classification of bodily sensation maps in fibromyalgia and control groups in Study 2.

Row-normalised confusion matrices are shown for the fibromyalgia group (A) and the control group (B), expressed as percentages of correctly and incorrectly classified trials for each true label. Each row represents the true category and each column represents the predicted category, such that values reflect the proportion of trials from a given category assigned to each predicted label. Diagonal elements indicate correct classifications, while off-diagonal elements reflect misclassifications between categories. The colour scale is matched across panels and normalised to the maximum percentage observed in the FM group to ensure comparability. White shading corresponds to performance below the theoretical chance level of 8%.

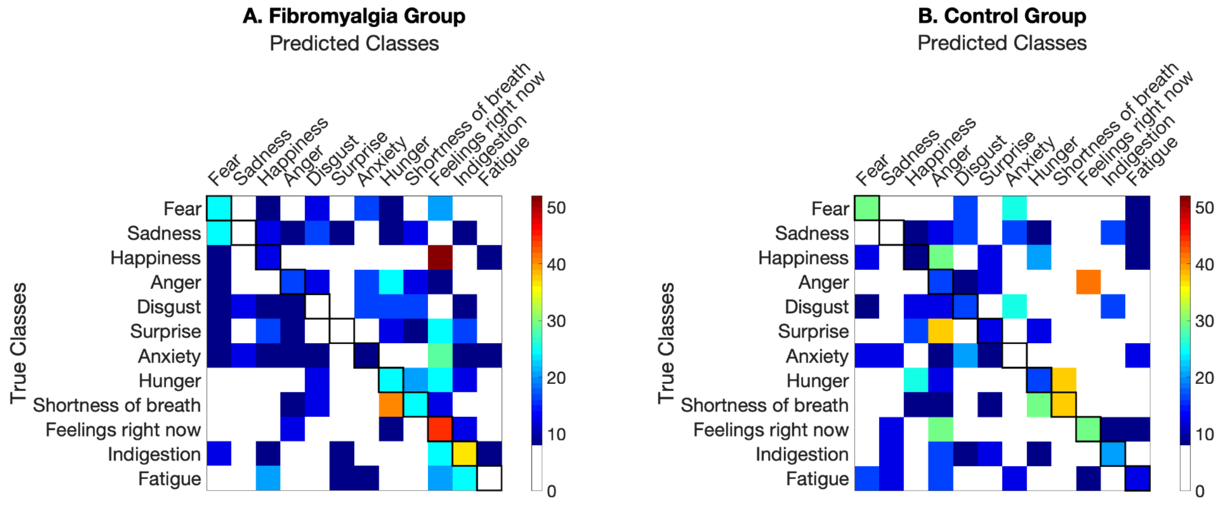

Supplement: Supplementary file 1 — Supplementary Material 1 [file 426_2026_2341_MOESM1_ESM.pdf]
